# Primary Open Angle Glaucoma and Vascular Risk Factors: A Review of Population Based Studies from 1990 to 2019

**DOI:** 10.3390/jcm9030761

**Published:** 2020-03-11

**Authors:** Andrzej Grzybowski, Mariusz Och, Piotr Kanclerz, Christopher Leffler, Carlos Gustavo De Moraes

**Affiliations:** 1 Institute for Research in Ophthalmology, Foundation for Ophthalmology Development, 61-285 Poznan, Poland; 2 Department of Ophthalmology, The Voivodal Specialistic Hospital in Olsztyn, 10-561 Olsztyn, Poland; mariuszoch@wp.pl; 3 Department of Ophthalmology, Hygeia Clinic, 80-286 Gdańsk, Poland; p.kanclerz@gumed.edu.pl; 4 Department of Ophthalmology, Virginia Commonwealth University, Richmond, Virginia, VA 23284, USA; chrislefflermd@gmail.com; 5 Bernard and Shirlee Brown Glaucoma Research Laboratory, Edward S. Harkness Eye Institute, Department of Ophthalmology, Columbia University Irving Medical Center, New York, NY 10032, USA; cvd2109@cumc.columbia.edu

**Keywords:** primary open angle glaucoma, diabetes, hypertension, migraine, population based study

## Abstract

Glaucoma is one of the leading causes of blindness worldwide, and as the proportion of those over age 40 increases, so will the prevalence of glaucoma. The pathogenesis of primary open angle glaucoma (POAG) is unclear and multiple ocular risk factors have been proposed, including intraocular pressure, ocular perfusion pressure, ocular blood flow, myopia, central corneal thickness, and optic disc hemorrhages. The purpose of this review was to analyze the association between systemic vascular risk factors (including hypertension, diabetes, age, and migraine) and POAG, based on major epidemiological studies. Reports presenting the association between POAG and systemic vascular risk factors included a total of over 50,000 patients. Several epidemiological studies confirmed the importance of vascular risk factors, particularly hypertension and blood pressure dipping, in the pathogenesis and progression of glaucomatous optic neuropathy. We found that diabetes mellitus is associated with elevated intraocular pressure, but has no clear association with POAG. No significant correlation between migraine and POAG was found, however, the definition of migraine varied between studies.

## 1. Introduction

Glaucoma is one of the leading causes of blindness worldwide [1]. As the proportion of those over age 40 increases, so will the prevalence of glaucoma. These demographics will challenge our resources and ingenuity [2]. Primary open angle glaucoma (POAG) is the most common form of the disease worldwide, particularly in Africa and in the Western countries [3,4,5]. POAG is defined as a progressive optic neuropathy with loss of ganglion cells and visual field deterioration in eyes with gonioscopically open angles, with or without elevated intraocular pressure (IOP). The pathogenesis of POAG is unclear. Multiple ocular risk factors have been proposed, including IOP, ocular perfusion pressure, ocular blood flow, myopia, central corneal thickness, and optic disc hemorrhages. Systemic risk factors include age, smoking, African ancestry, family history, genetic factors, systemic hypertension (HTN), low blood pressure (BP) (particularly a nocturnal drop in BP), atherosclerosis, lipid dysregulation, type 2 diabetes mellitus (DM), glucose intolerance, obesity, vasospasm, migraine, Raynaud syndrome, stress, and primary vascular dysregulation [5,6,7,8,9,10,11,12]. Genetic abnormalities are believed to initiate a cascade of events that lead to glaucomatous optic nerve injury and remodeling. Several studies have revealed a significant role of the myocilin, optineurin, and cytochrome CYP1B1 genes in glaucoma development [13,14,15,16]. Moreover, genome-wide association studies have shown associations of sequence variants [17,18,19,20,21] and single-nucleotide polymorphisms [22,23,24,25,26] with POAG. Notwithstanding the role of genetic mutations and variations on glaucoma onset, the role of different systemic risk factors remains debatable.

The purpose of this study was to analyze the association of systemic vascular risk factors (including HTN, diabetes, age, and migraine) and POAG, based on major epidemiological studies.

## 2. Materials and Methods

### 2.1. Literature Search

We used the PubMed database to identify studies that included the term glaucoma in association with the following keywords (Appendix A)—vascular risk factors, systemic risk factors, blood pressure, systemic hypertension, systemic hypotension, diabetes, dipping, vasospasm, age, and migraine. The search identified 270 unique articles. Only English-language articles were selected. Articles cited in the reference lists of other publications and review articles were also considered for inclusion. Studies were critically reviewed to create an overview and guidance for further search. No attempts to discover unpublished data were made. Articles providing original research were included. We also incorporated information from relevant reviews and meta-analyses.

### 2.2. Study Selection

We identified all studies evaluating the association between the selected risk factors and POAG. Articles were included in our meta-analyses if they met the following criteria—(1) the study design was a population-based study; (2) the association of the exposure of interest with POAG was evaluated; (3) the outcome of interest was POAG; and (4) risk ratio (RR), odds ratio (OR), hazard ratio (HR), or proportions and their corresponding 95% confidence interval (CI) (or data to calculate them) were reported. If more than one identified article reported on the same study population, we selected the study with the longest follow-up or the most recent study. We assessed the risk of bias using the methods described by Sanderson et al. [27]; we evaluated the methods for selecting study participants, various study designs, and criteria for defining exposures and outcomes. Studies were included in our meta-analyses if they provided quantitative estimates of POAG prevalence in population-based surveys. We excluded research evaluating association between vascular risk factors and normal tension glaucoma. Investigations were not excluded on the basis of clinical definitions of POAG or methods used to diagnose POAG cases; studies using self-reported diagnosis of glaucoma were excluded. Articles that did not meet the aforementioned criteria were not included in the meta-analysis, however, could be presented within the discussion.

### 2.3. Data Extraction

Two authors (A.G. and M.O.) separately reviewed all searched articles to determine the eligible studies and the extracted data from the selected results. Any disagreements were resolved by adjudication. The data extraction was performed using a standardized data-collection form. Information was extracted as follows—the first author’s last name; publication year; country location; characteristics of study population (age, number of participants); number of POAG patients; methods for identification of risk factors for glaucoma in question; RR (or OR) from the most fully adjusted models and its corresponding 95% CI; and statistical adjustments for the confounding factors.

### 2.4. Statistical Analysis

Statistical calculations were conducted with the Stata package (Special Edition, release 14.2, StatCorp LLC, College Station, TX, USA). The meta-analysis was performed as recommended by the Cochrane Collaboration [28]. In order to present the data visually, forest and funnels plots were created. Forest plots are routinely used in meta-analysis, while funnel plots are a good way to show publication bias; funnel plot asymmetry might reflect differences in the results between studies, or bias of particular studies. Inconsistency was measured with the I^2^ function [29,30]. A quantity of the I^2^ equal to 0% indicated no observed heterogeneity, while 100% indicated no consistency of the analyzed data.

## 3. Results

We collected and analyzed 88 articles dated from 1993 to 2019. Most of the included studies were conducted over the last decade. Studies presenting the association between POAG and systemic vascular risk factors included a total of over 50,000 patients (Table 1). Only one study assessed all of the analyzed risk factors [31]. Other studies investigated HTN (13 studies), diabetes (12 studies), age (5 studies), and migraine (4 studies). The meta-analyses encompassed eighteen original papers and the results were presented on forest plots.

**Figure 1:**
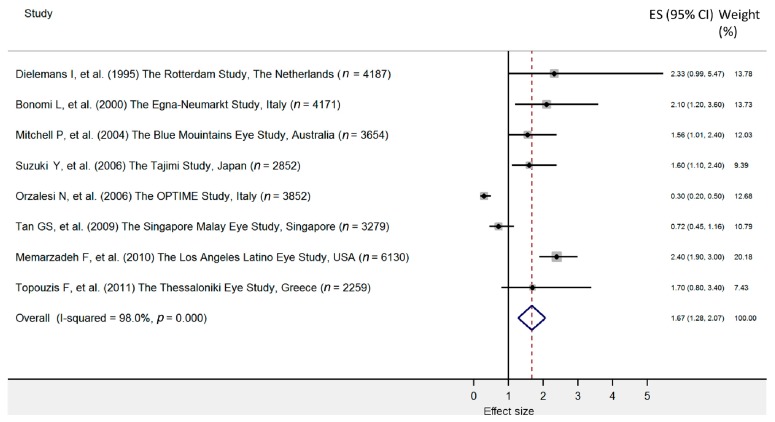
Forest plot of cohort studies that evaluated arterial hypertension as a risk factor for primary open angle glaucoma. The effect size (square, with size proportional to the weights used) and 95% confidence intervals (horizontal lines) is presented for each study. The overall measure revealed by pooled analysis is marked with the center of the diamond, while the associated confidence intervals are indicated by the lateral tips of the diamond [31,33,35,40,42,46,49,50].

**Figure 2:**
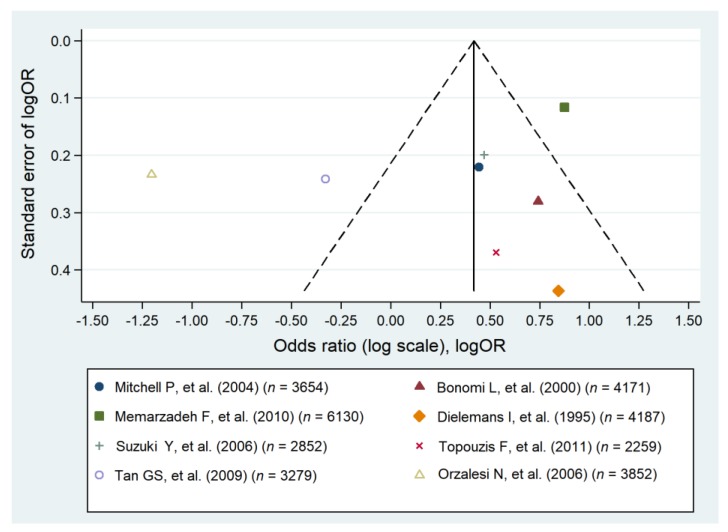
Funnel plot of cohort studies that evaluated arterial hypertension as a risk factor for primary open angle glaucoma [31,33,35,40,42,46,49,50].

Most of the analyzed studies identified an association of HTN with glaucoma and optic nerve damage [31,33,35,40,46,48,49,50,52] (Table 1). The meta-analysis involving eight original studies on the prevalence of arterial HTN revealed a significant relationship with the occurrence of POAG (pooled OR = 1.67; 95% CI: 1.28–2.07) (Figure 1). Despite the high heterogeneity of our results (I^2^ = 98.0%), it could be concluded that hypertension is associated with POAG (Figure 2).

**Figure 3:**
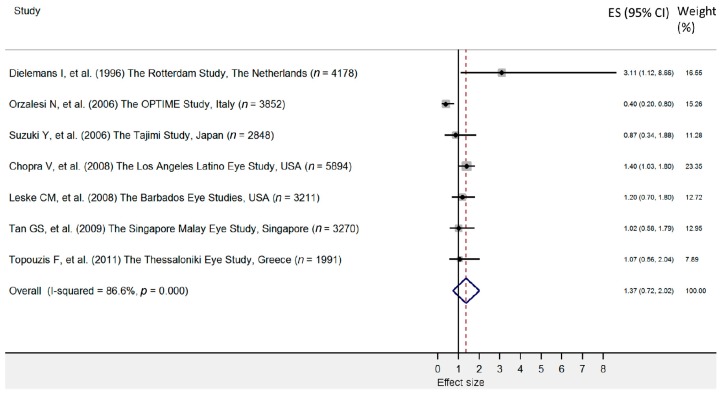
Forest plot of cohort studies that evaluated diabetes as a risk factor for primary open angle glaucoma. The effect size (square, with size proportional to weights used) and 95% confidence intervals (horizontal lines) is presented for each study. The overall measure revealed by pooled analysis is marked with the center of the diamond, while the associated confidence intervals are marked by lateral tips of diamond [31,32,34,42,46,49,51].

**Figure 4:**
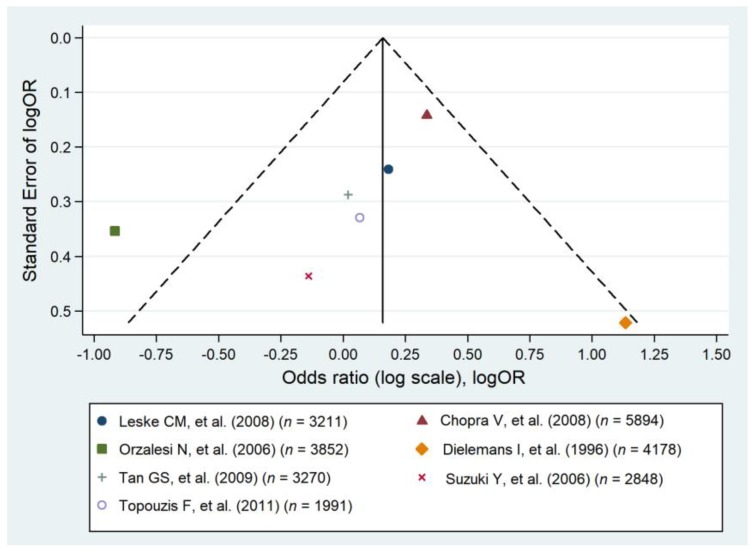
Funnel plot of cohort studies that evaluated diabetes as a risk factor for primary open angle glaucoma [31,32,34,42,46,49,51].

It was reported that DM is associated with an elevated IOP [53]; therefore, DM influence POAG by mediation of IOP. However, current evidence to support a direct relationship remains conflicting and is still not fully understood [6]. Seven population-based studies reported a statistically significant association between DM and POAG [34,38,41,46,48,49,51], but five studies did not confirm an association [31,32,36,42,43]. In the pooled analysis comprising seven original studies no significant relationship between the prevalence of POAG and DM was identified (pooled OR = 1.37; 95% CI: 0.72–2.02), and heterogeneity was high (I^2^ = 86.6%) (Figure 3 and Figure 4).

All studies that investigated the correlation between age and POAG confirmed this association [31,32,45,46,47]. Studies assessing the POAG prevalence and patient age approached the issue in very different ways. Various age ranges were assessed; some studies presented ORs [31,33,34,35,36,38,40,41,42,44,46,48,49,50,51], others provided RRs [32,47,54] or HRs [45], while some reported proportions and their corresponding 95% CIs [37,43]. A number of studies did not provide the effect sizes. There were also original articles in which age was not evaluated as an independent variable. In order to perform a reliable meta-analysis considering the prevalence of POAG versus age, the individual study results must be coherent, i.e., contain the same effect size measures (either OR or RR), and identical class intervals for the patients’ age. Given the variable quality and heterogeneity of the reviewed studies, and that the results focused upon the relationship bet ween age and prevalence of POAG, a meta-analysis was not performed.

**Figure 5:**
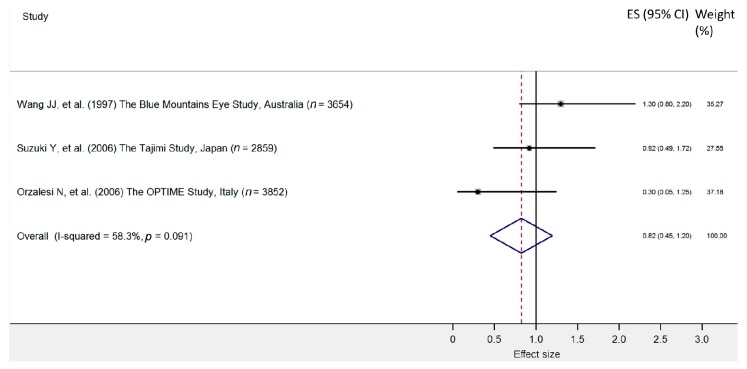
Forest plot of cohort studies that evaluated migraine as a risk factor for primary open angle glaucoma. The effect size (square, with size proportional to weights used) and 95% confidence intervals (horizontal lines) is presented for each study. The overall measure revealed by pooled analysis is marked with the center of the diamond, while the associated confidence intervals are marked by the lateral tips of the diamond [11,31,49].

**Figure 6:**
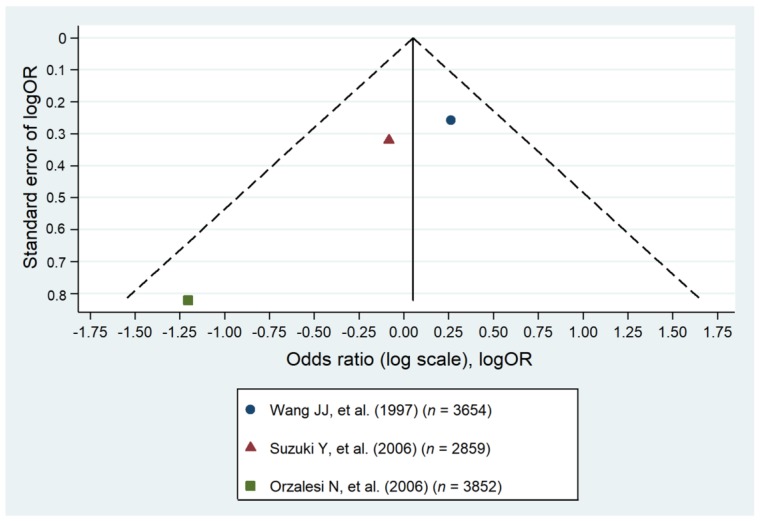
Forest plot of the cohort studies that evaluated migraine as a risk factor for the primary open angle glaucoma [11,31,49].

One study suggested an association between migraine and glaucoma among patients in the age range of 70 to 79 [11]. Three studies identified no association between glaucoma and migraine [31,39,49]. Our meta-analysis encompassing three original studies identified no association between glaucoma and migraine (pooled OR = 0.82; 95% CI: 0.45–1.20). As the heterogeneity was low, the results can be accepted with greater certainty (Figure 5 and Figure 6).

## 4. Discussion

### 4.1. Systemic Hypertension and Hypotension

The majority of studies defined HTN as systolic BP (SBP) greater than 160 mm Hg, diastolic BP (DBP) greater than 95 mmHg, or the use of anti-HTN medications ascertained from interview with patients. The study by Topouzis et al. found that both treated HTN (with BP within normal limits), and high systolic or diastolic BP are risk factors for POAG, with an OR for the presence of POAG being 2.04 (95% CI: 0.88–4.73) and 1.43 (95% CI 0.70–2.91), respectively [31,46]. Moreover, the severity of glaucoma was found to be higher in patients with HTN than in glaucomatous individuals with a normal BP [55]. The associations of SBP and DBP with glaucomatous optic neuropathy depend on the specifics of the patients studied—age, ethnic origin, concurrent diseases, other risk factors, treatment of HTN, type of medication used, and vascular endothelial function. Several mechanisms have been suggested to explain the risk of the developing POAG in individuals with HTN [56]. Abnormal neuro-metabolic-endothelial mechanisms of the vascular tone control in glaucoma patients is related to the effects of vasoactive factors—endothelin–1 (ET-1), neuropeptide Y (NPY), nitric oxide (NO), prostacyclin (PGI 2), tumor necrosis factor (TNF-alpha), cyclooxygenase (COX-2), and metalloproteinases (MMP-9). Variation in the levels of these factors leads to endothelial dysfunction and to dysregulation of the autonomic nervous system. These abnormalities can produce vasoconstriction and optic nerve head ischemia in normal tension glaucoma (NTG) [3]. Theoretically, the duration of HTN and degree of vascular damage could be correlated with the risk of glaucoma; however, none of the studies analyzed such an association. A study by Jung et al. found that glaucomatous patients with multiple retinal nerve fiber layer (RNFL) defects had a higher prevalence of concomitant vascular diseases (including HTN, end-stage renal disease or cerebrovascular disease) compared to those with glaucoma and no RNFL defects [57]. Additionally, HTN was found to be a risk factor for reduced RNFL in healthy individuals in the European Eye Epidemiology study [58]. These results highlight the potential role of vascular insufficiency, not only as a risk factor of glaucoma, but also for potential glaucomatous damage [57]. Moreover, smoking was found to be a risk factor for POAG, and smoking presumably deteriorated ocular hypoxia caused by the ocular hypertension [59]. The analyzed studies did not determine smoking as a risk factor for POAG incidence [46] or progression [43]. However, heavy smoking, which was defined by a greater number of cigarette packs smoked daily was associated with higher odds of glaucoma [60]. With this, the role of cytochrome CYP1B1 was confirmed in primary congenital glaucoma, as it influenced the development of the trabecular meshwork [61,62]; CYP1B1 is also a potential regulator in energy homeostasis that promotes hypertension as well [63].

Systemic BP has a circadian variation [8] and a growing number of studies analyzed the ocular perfusion pressure, defined as the difference between systemic pressure and IOP. Current evidence suggests that ocular perfusion pressure and IOP variations have a detrimental effect in the glaucomatous eye [64]. In patients with glaucoma and well-controlled HTN, a nocturnal BP fall of more than 10% was associated with a greater visual field defect and a greater degeneration of optic nerve fibers [65]. Nocturnal hypotension, in the presence of other vascular risk factors, might reduce the optic nerve head blood flow below a critical level and might play a role in the pathogenesis of glaucomatous optic neuropathy [66,67]. Another study also suggested that non-physiological nocturnal BP dipping and wider circadian fluctuations in ocular perfusion pressure were linked with the development and progression of glaucoma [8]. In the Maracaibo Aging Study, extreme decreases in nighttime systolic and diastolic BP (defined as a BP decrease greater than 20%, compared to daytime levels) were significant risk factors for glaucomatous damage (with the odds ratio 19.78 and 5.55, respectively) [68]. On the other hand, Yoshikawa et al. found that individuals with glaucoma have higher nighttime systolic BP (by 4.1 mmHg) than the healthy controls [69]. It was suggested that glaucoma progression in such cases is associated with insufficient vascular autoregulation [70]. Patients with POAG and those with NTG exhibit similar increases in nocturnal systemic BP variability, peripheral arterial stiffness, and carotid intima-media thickness, as well as reduced ocular perfusion pressure [71]. According to Flammer et al. both excessive nocturnal BP drop (over-dipping), or absent BP drop (non-dipping) are risk factors for glaucoma progression [70]. Moreover, low systemic DBP itself was defined as a risk factor for glaucoma progression [42,50].

**Figure 7:**
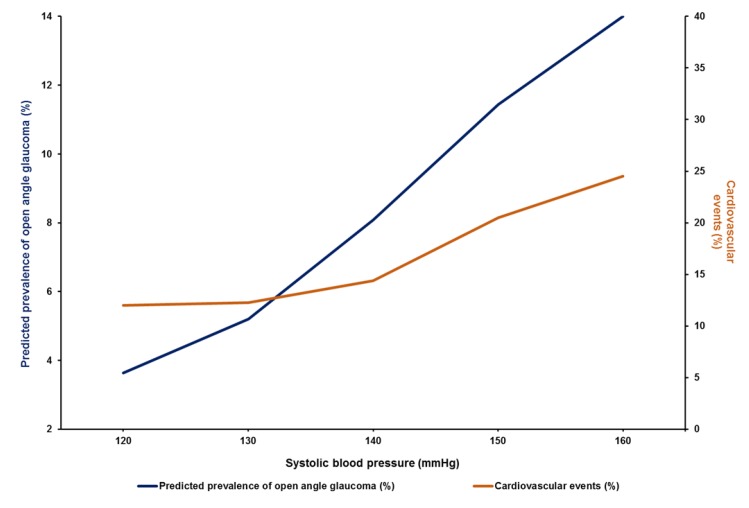
The relationship between SBP and the predicted prevalence of primary open angle glaucoma (POAG) in participants in the Los Angeles Latino Eye Study and cardiovascular events in the Ontarget study [50,73].

**Figure 8:**
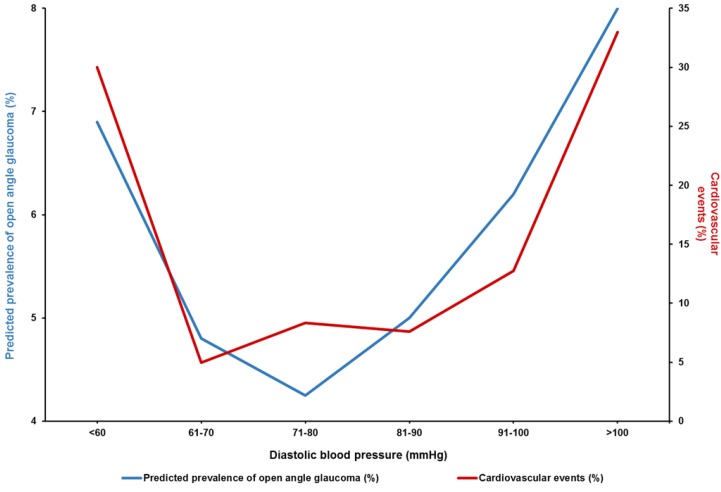
The relationship between diastolic BP (DBP) and the predicted prevalence of POAG in participants of the Los Angeles Latino Eye Study and cardiovascular events in the TNT study [50,74].

Abnormally low diastolic double product (dDP = DBP × heart rate) can be another marker of cardiovascular autonomic dysregulation, resulting in low ocular perfusion in NTG patients [72]. A positive correlation between SBP and the predicted prevalence of open angle glaucoma was reported in the Los Angeles Latino Eye Study [50]. A similar influence of SBP on cardiovascular risk factors was shown by the cardiovascular Ontarget trial (Figure 7) [73]. This study aimed to analyze the impact of systemic BP on cardiovascular events in well-treated, high-risk patients, enrolled in a large clinical trial. The analysis revealed that very high SBP was associated with glaucoma; moreover, a clear relationship between DBP and the predicted prevalence of OAG was observed (Figure 8) [50]. A very similar relation between DBP and cardiovascular risk (a so-called J-curve) was also observed in Coronary Artery Disease (CAD) patients, in the TNT study [74]. The TNT study showed that in patients with CAD, high DBP (>90–100 mmHg) and low DBP (<60–70 mmHg) portend an increased risk of future cardiovascular events. Randomized controlled trials of antihypertensive treatment provide strong evidence for J-shaped relationships between both DBP and SBP, and mortality (including all-cause mortality, cardiovascular mortality, nonfatal and fatal myocardial infarct, heart failure, stroke) in high-risk cardiovascular populations, including patients with CAD, DM, and left ventricular hypertrophy [75]. A similar J-shaped association was found between BP and glaucoma progression; patients with both high and low BP were at risk of glaucoma progression [76]. The treatment of HTN could also influence the risk of POAG; oral treatment with beta-blockers was shown to decrease the risk of POAG [77], while intake of calcium channel blockers (and particularly amlodipine) was associated with glaucoma severity and was found to increase the risk of POAG that required surgical intervention [78].

### 4.2. Diabetes Mellitus

DM is associated with microvascular damage and might affect vascular autoregulation of the retina and the optic nerve [51]. DM is not a homogenous condition. Study populations have varied with respect to diabetes characteristics—the time since diagnosis, type of treatment (diet, oral medication, insulin) as well as the presence of various complications from diabetes. The aforementioned studies did not control for the type of diabetes or the treatments used [31,32,34,36,38,41,42,43,46,48,49,51].

Many hypotheses have been offered to explain the correlation between DM and POAG. The first theory suggests that long-standing hyperglycemia and lipid dysregulation might increase the risk of neuronal stress damage [79]. A second theory proposes that diabetic eyes have a dysregulation in blood flow due to retinal vascular endothelial cell dysfunction; this hypothesis was confirmed both in animal studies and subsequent clinical trials [80]. Another explanation for the association between DM and POAG might be a remodeling of the connective tissue of the optic nerve head and dysregulation at the trabecular meshwork and the lamina cribrosa, with increased IOP and greater mechanical stress on the optic nerve head. Diabetes can exacerbate connective tissue remodeling and amplify these biomechanical changes [81,82]. Vascular endothelial cell dysfunction and loss of retinal pericytes have been described in diabetic retinopathy and are associated with hypoxia [83]. Thus, the function of the endothelium could theoretically be a marker of proper nourishment of the optic nerve cells. Moreover, the anatomical and functional status of the vessels can influence the structure and function of the optic nerve. Additionally, metabolic disturbances of the carbohydrate metabolism could play a role in glaucoma damage and pathogenesis [84]. Finally, diabetes is associated with an increased IOP, and this association is not through the influence of diabetes of central corneal thickness [85]. The aforementioned factors could highlight the importance of vascular risk factors in the pathogenesis of POAG. Interestingly, patients with POAG and treated DM tend to have significantly lower rates of RNFL thinning compared to those without diagnosed DM [86].

Earlier meta-analyses suggested that diabetic patients are at a significantly increased risk of developing POAG [87,88,89]. In a study by Zhao et al. the risk of glaucoma increased by 5% for each year since diabetes diagnosis; their pooled analysis presented a 0.18 mmHg difference between IOP in patients with diabetes, compared to those without diabetes [90]. Our study identified no significant relationship between the prevalence of POAG and diabetes mellitus; the high heterogeneity among studies and, thus, the conclusion, must be interpreted with caution. Results for case-control studies could be different, e.g., in a Korean study, diabetes was associated with POAG in all age groups (the HR was 1.2 for individuals aged 40–59 years, and 1.18 for those aged 60–79 years) [91]. However, it is known that case-control designs are potentially viable to selection bias [92].

### 4.3. Age

The prevalence of glaucoma varies substantially by geographical region, ethnicity, and age group. The incidence of POAG ranges from 0.8% (in the Caucasian population in the Barbados Eye Study) [93] to 4.4% (African-descent population in the Barbados Eye Study) [32]. The prevalence of glaucoma is the highest in elderly patients. Even greater prevalence rates in the elderly were reported in people of African descent—11% in those aged over 80 years in the Baltimore Eye Survey [37], 23.2% in the patients over 75 years of age in the Salisbury Eye Evaluation Glaucoma Study [94], and 24.8% in the male population aged 80 to 86 years in the Barbados Eye Study [93]. Additionally, a recent database analysis study confirmed a strong relationship between age and the prevalence of POAG, however, the incidence rates increased until the age of 85–89 years, and then slightly decreased in those over 90 years of age [95].

There could be several explanations why the prevalence of glaucoma increases with age. First, experimental studies have shown that with age there is a reduction in trabecular lumen area and a subsequent decrease in aqueous outflow [96]. Second, biomechanics of the eye and optic nerve changed considerably with aging; it was proposed that the aged optic nerve has a more stiffened peripapillary scleral and lamina cribrosa connective tissue, and thus, the optic nerve head is more susceptible to axonal damage [97]. Moreover, as there is a high density of mitochondria around the optic disc, age-related mitochondrial dysfunction would reduce the energy supply [98,99]. Finally, aging-induced retrobulbar hemodynamic changes are similar to those seen in glaucoma; thus vascular changes could contribute to an increased risk for glaucoma in older age [9].

### 4.4. Migraine

Both migraine and glaucoma are associated with systemic vascular dysregulation or vasospasm [7]. Moreover, as migraine is accompanied with persistent blood flow changes in several brain regions [100], similarly poor blood flow at the optic nerve was reported in glaucoma [101]. Our meta-analysis demonstrated no significant relationship between migraine and the prevalence of POAG. However, the Collaborative Normal-Tension Glaucoma Study Group analyzed the risk factors for progression of visual field abnormalities in NTG and found migraine to be an independent risk factor for more rapid deterioration [102]. Moreover, migraineurs are more likely to develop POAG compared to subjects without migraine (95% confidence interval of POAG incidence: 1.20–2.36) [103]. Age, hyperlipidemia, and DM were noted as three significant risk factors for developing POAG in migraineurs [103]. Our meta-analysis encompassing three original studies identified no association of glaucoma and migraine. Similar results were reported in a recent meta-analysis by Xu et al.; however, one part of their analysis which included case-control studies found a significant association between migraine and POAG [104].

It should be underlined that the definition of migraine differed among studies. For example, in the Beaver Dam Eye Study, which presented no association between glaucoma and migraine, the question used for migraine diagnosis might have been overly restrictive—“Have you ever had migraine headaches (with vomiting, light flashes, or severe enough to keep you in bed) [39]?” Moreover, in the included studies migraine was only analyzed in a post-hoc fashion.

## 5. Conclusions

Several epidemiological studies confirmed the importance of vascular risk factors in the pathogenesis and progression of glaucomatous optic neuropathy, including hypertension and BP dipping. The pooled analysis confirmed hypertension as the most significant risk factor for primary open angle glaucoma. The association between POAG and diabetes mellitus type 2 or migraine still needs to be evaluated in future studies.

## Figures and Tables

**Table 1:** Studies investigating systemic vascular risk factors in primary open angle glaucoma.

Study	Country (Population)	Study Design	Participants (*n*)	Patients’ Age	Statistically Significant Association with				
					Systolic Hypertension	Low DBP	Diabetes Mellitus	Age	Migraine
Barbados Eye Study [32]	Barbados (African descent)	Population-based cohort study (prospective)	3222	40–84	No (*p* = 0.26)	Yes	No (*p* = 0.49)	Yes (*p* = 0.0001)	N/A
Rotterdam Study [33,34]	The Netherlands	Population-based cross-sectional study (prospective)	4187	55+	Yes	No	Yes	NA	NA
Egna Neumarkt study [35]	Italy	Population-based cross-sectional study	4297	40+	Some association	Yes (for low DPP)	NA	NA	NA
Baltimore Eye Survey [36,37]	USA	Population-based prevalence survey	5308	40+	Yes (SBP and DBP)	Yes for low DPP	No	NA	NA
Beaver Dam Study [38,39]	USA	Population based study.	4926	43–84	NA	NA	Yes (*p* = 0.004)	NA	No (*p* = 0.87)
Blue Mountains Eye Study [11,40,41]	Australia	Population-based survey	3654	49–96	Yes	Some association (*p* = 0.05 when Age and Sex Adjusted)	Yes	NA	Yes (in 70–79 years of age)
The Singapore Malay eye study [42]	Singapore (Malay)	Population-based cross-sectional study	3280	40–80	No	No	No	NA	NA
The Beijing Eye Study [43,44]	China	Population-based survey	3251	40+	No risk for glaucoma progression	No risk for progression of glaucoma	No	NA	NA
Tajimi Study [31]	Japan	Population-based cross-sectional study	2874	40+	Yes (*p* = 0.0094)	NA	No (*p* = 0.85)	Yes (*p* < 0.0001)	No (*p* > 0.99)
Early Manifest Glaucoma Trial [45]	Sweden	Randomized clinical trial	255	50–80	No risk for progression of glaucoma (*p* = 0.1279)	Yes (risk for progression of glaucoma in patients with low baseline IOP)	NA	Yes (*p* = 0.0270)	NA
Thessaloniki Eye trial [46]	Greece	Population-based cross-sectional study	1991	60+	Yes (*p* = 0.033)	NA	Yes (*p* = 0.003)	Yes (*p* = 0.001)	NA
Visual Impairment Project [47]	Australia	Cohort study	2415	40+	NA	NA	NA	Yes	NA
African eyes [48]	Nigeria	Hospital-based multicenter study (prospective)	506	10+	Yes	No	Yes	NA	NA
Optime study [49]	Italy	Observational survey	3852	55+	Yes	No	Yes	NA	No
Los Angeles Latino Eye Study [50,51]	USA (Latino)	Population-based cross-sectional study	6130	40+	Yes	No	Yes	NA	NA

DBP, diastolic blood pressure; DPP, diastolic perfusion pressure; DPP = DBP − intraocular pressure. NA, not applicable; SBP, systolic blood pressure; *p* values are presented if provided in the study.

## Appendix A. Method of Literature Search

The literature was searched on the MEDLINE database using PubMed, for articles in English, published between the 1990 and 2019, using the keywords: “vascular risk factors”, “systemic risk factors”, “blood pressure”, “systemic hypertension”, “systemic hypotension”, “diabetes”, “dipping”, “vasospasm”, “age”, and “migraine”. All returned articles, as well as their references, were screened for relevant data on the association between the selected risk factors and POAG. We included only population-based studies in which the exposure of interest and POAG was evaluated. We excluded studies using self-reported diagnosis of glaucoma. The reference lists of relevant papers were reviewed to identify additional papers, this process was iterated several times.
